# Hemodynamic Impact of Morphological Changes in Intracranial Aneurysms Treated with the Contour Neurovascular System: A Pilot In Silico Study

**DOI:** 10.3390/brainsci16080830

**Published:** 2026-08-04

**Authors:** Anna Bernovskis, Fina Gießler, Franziska Gaidzik, Sylvia Saalfeld, Naomi Larsen, Philipp Berg

**Affiliations:** 1 Department of Medical Engineering, Otto von Guericke University Magdeburg, 39106 Magdeburg, Germany; 2 Research Campus STIMULATE, Otto von Guericke University Magdeburg, 39106 Magdeburg, Germany; 3 Institute for Medical Informatics and Artificial Intelligence, University Hospital Schleswig-Holstein, 24105 Kiel, Germany; 4 Department of Fluid Dynamics and Technical Flow, Otto von Guericke University Magdeburg, 39106 Magdeburg, Germany; 5 Department for Radiology and Neuroradiology, University Hospital Schleswig-Holstein, 24105 Kiel, Germany

**Keywords:** Contour Neurovascular System, endovascular treatment, image-basedblood flow simulation, intrasaccular device, post-interventional data

## Abstract

**Background**: The Contour Neurovascular System (CNS) has been established as an effective treatment for wide-neck intracranial bifurcation aneurysms. However, its post-intervention hemodynamic impact remains insufficiently understood. In silico studies investigating the associated hemodynamic changes are limited and rarely consider post-interventional configurations. **Methods**: Five intracranial aneurysms treated with the CNS were retrospectively analyzed using both pre-interventional and 24 h post-interventional imaging data. CNS-associated morphological changes were assessed, and virtual CNS deployment was performed in pre- and post-interventional configurations. Consequently, 20 high-fidelity, image-based blood flow simulations were performed to evaluate six flow- and shear-related parameters. **Results**: All cases exhibited an increase in ostium area between pre- and post-interventional configurations (118.7% ± 52.4%), whereas no relevant geometric differences were observed in the surrounding vessels. Hemodynamic analyses revealed that morphological differences were associated with variations in flow characteristics. In particular, most post-interventional configurations showed higher intra-aneurysmal velocities independent of device implantation, although CNS deployment consistently reduced all investigated parameters (reduction in intra-aneurysmal velocity—pre-interventional with CNS: −83.5% ± 14.0%; post-interventional with CNS: −72.4% ± 19.4%). **Conclusions**: In this exploratory study, the observed CNS-associated morphological differences were accompanied by variations in aneurysmal hemodynamics, potentially influencing the estimated treatment effect. Although validation in larger cohorts is required, these findings highlight the potential value of 3D-DSA follow-up imaging for a more comprehensive assessment of CNS-associated changes and outcomes.

## 1. Introduction

The Contour Neurovascular System (CNS, Stryker, Portage, USA) has emerged as a promising endovascular treatment option for intracranial aneurysms (IAs), demonstrating favorable occlusion rates and encouraging clinical outcomes in several studies [1,2,3,4]. Nevertheless, recent investigations have reported cases of device deformation during follow-up, raising concerns regarding its potential impact on long-term treatment success and aneurysm recurrence [5,6]. While these observations highlight the need for further research into device stability, it remains unclear whether CNS implantation directly influences vascular remodeling or whether vessel deformation contributes to subsequent changes in device configuration.

Understanding these interactions is particularly important because aneurysm healing is a dynamic process involving thrombus formation, endothelialization, and vascular remodeling [7]. Consequently, post-interventional configurations may differ substantially from the pre-interventional configurations that are commonly used for computational analyses. Such morphological changes could alter local hemodynamics and thereby influence treatment outcomes.

Patient-specific numerical blood-flow simulations have become an established tool for investigating the hemodynamic mechanisms underlying aneurysm treatment [8,9]. However, the use of post-interventional imaging data presents several challenges. In clinical routine, follow-up imaging is often acquired using modalities other than the gold-standard three-dimensional digital subtraction angiography (3D-DSA) [10,11], resulting in reduced image quality and geometric inaccuracy. Furthermore, the CNS is specifically designed to reduce blood flow into the aneurysm sac [4]. The resulting decrease in intra-aneurysmal contrast agent concentration can complicate image segmentation and may lead to an incomplete representation of the aneurysm dome in post-interventional configurations. These limitations have contributed to the widespread practice of performing hemodynamic simulations solely on pre-interventional configurations with virtually deployed devices [12,13,14].

However, the validity of this approach remains uncertain when substantial morphological changes occur after treatment. Therefore, the present study analyzes post-interventional configurations following CNS implantation. Specifically, the objectives are as follows:

- To analyze morphological changes between pre-interventional and post-interventional configurations.
- To investigate the influence of these morphological changes on aneurysm hemodynamics.

By comparing simulations based on pre-interventional and post-interventional configurations, this exploratory study evaluates whether the commonly used pre-interventional modeling approach is sufficient for accurately characterizing CNS-associated hemodynamics and device performance, while providing the first assessment of the hemodynamic impact of morphological changes induced by CNS deployment.

## 2. Materials and Methods

### 2.1. Patient Information

Five cases of bifurcation aneurysms treated at the University Medical Center Kiel (Schleswig-Holstein, Kiel, Germany) using a CNS device were retrospectively analyzed in this study. The interval between pre-interventional 3D-DSA and post-interventional computed tomography angiography (CTA) was around 24 h.

Pre-interventional 3D-DSA datasets were acquired using a biplane angiography system (Azurion, Philips, Amsterdam, The Netherlands). The reconstructed voxel size for 3D-DSA was 0.16 mm isotropic. Post-interventional CTA examinations were performed on a Spectral CT 7500 system (Philips, Amsterdam, The Netherlands), with reconstructed voxel dimensions of 1.0 × 0.35 × 0.35 mm. Device selection was performed according to the institutional clinical workflow. Pre-interventional 3D-DSA data were visualized using the manufacturer’s vessel analysis application as a semi-transparent three-dimensional surface reconstruction. The maximum aneurysm neck width was measured manually in the ostium plane. The maximum aneurysm dome width was measured in two orthogonal directions on a plane parallel to the ostium plane, and the average of these measurements was used for device sizing. Based on these measurements, device selection followed the manufacturer’s sizing recommendations (in Appendix A.1, Table A1). If the aneurysm dimensions did not match one of the predefined sizing categories, the next larger device size was selected. Detailed patient and aneurysm characteristics are provided in Table 1. The occlusion status was assessed on the 24 h post-interventional angiography and the longest available follow-up imaging using the Bicêtre Occlusion Scale Score (BOSS) [15]. All aneurysms showed favorable angiographic outcomes at follow-up, with complete occlusion or a stable neck remnant, except for IA2, which demonstrated a small neck remnant (BOSS 1).

### 2.2. Model Preparation

The medical images were segmented using a region-growing algorithm implemented in MeVisLab (v3.4.2; Fraunhofer MEVIS, Bremen, Germany). To ensure physiologically realistic flow development for the numerical simulations, the inlet and outlets were extruded using Blender (v4.2, Blender Foundation, Amsterdam, The Netherlands). In addition, artifacts were manually removed. For the post-interventional configurations, segmentation of the IA region proved to be considerably more challenging. This is primarily due to the lower image quality and the reduced blood flow within the aneurysm dome following device placement, resulting in lower contrast agent visibility in this region. In previous studies regarding post-interventional configurations, a “stitching” approach is often employed, where the aneurysm dome from the pre-interventional configuration is transferred onto the vessel geometry of the post-interventional configuration [13,16,17]. However, this method requires substantial adjustments and may introduce additional sources of uncertainty. Therefore, this study avoided such a stitching approach. Instead, the post-interventional configuration was segmented directly from the available imaging data, including only the contrast-enhanced lumen that could be reliably identified in the post-interventional images. This direct segmentation approach was chosen to minimize artificial alterations to the post-interventional configuration and to preserve the geometric information actually contained in the imaging data. The strategy was selected after consultation with an experienced neuroradiologist and evaluation of different reconstruction approaches. While this approach avoids artificial alterations to the post-interventional configuration, complete reconstruction of the aneurysm sac was no longer feasible. Another challenge arises from the use of different imaging modalities for the pre-interventional and post-interventional datasets. Therefore, the corresponding 3D models were additionally registered in MeshLab (v2023.12, Visual Computing Lab, Pisa, Italy) and subsequently cropped to the desired region of interest. The geometric differences between the resulting models were then quantitatively analyzed using MATLAB (vR2024b; MathWorks, Natick, MA, USA). A detailed description of the workflow and the corresponding results is provided in Appendix A.2 and referenced in Section 4. For ostium extraction and the computation of morphological parameters, a MATLAB script developed by Saalfeld et al. [18] was used. The required vessel centerlines were generated using the open-source Vascular Modeling Toolkit (VMTK) [19]. This semi-automatic approach reconstructs the aneurysm neck curve based on the vessel centerline and predefined geometric criteria, thereby reducing potential operator-dependent variability in ostium delineation.

#### Virtual CNS Deployment

**Figure 1:**
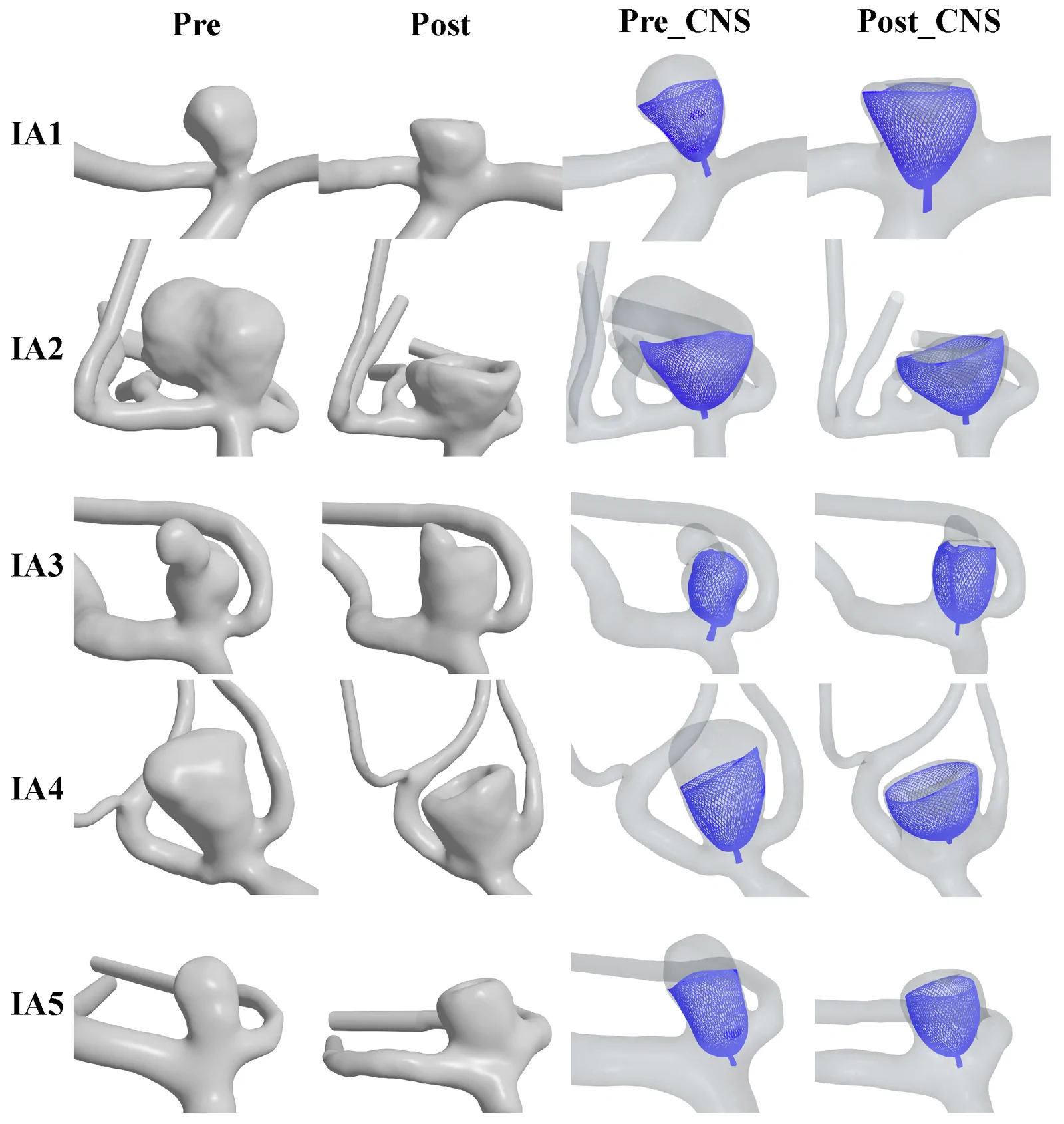
Overview of the investigated aneurysms, illustrating the pre-interventional and post-interventional configurations. For each case, both anatomical configurations are shown without and with the virtually deployed CNS, yielding four representations per aneurysm: pre-interventional configuration, post-interventional configuration, pre-interventional configuration with virtual CNS deployment, and post-interventional configuration with virtual CNS deployment.

For both pre- and post-interventional configurations, the CNS was virtually deployed. Computer-Aided Design models of different CNS sizes were provided by the manufacturer. In post-interventional configurations, the position of the CNS could be inferred from medical imaging data, whereas in pre-interventional configurations, the position was determined in consultation with a neuroradiologist to ensure realistic scenarios. The device sizes corresponded to those used during the procedure. The CNS was placed using the open-source software Blender, v4.2 where it was adapted and positioned according to the individual anatomy using the lattice modifier. This approach is currently considered one of the most suitable methods for virtual deployment of CNSs and has been validated in previous studies, including a combined in vitro and in silico study to ensure correct placement [17,20,21]. An overview of the segmented models and the virtually deployed CNS is shown in Figure 1.

### 2.3. Image-Based Blood Flow Simulations

For each case and configuration, unsteady image-based blood flow simulations were performed using the finite-volume-based solver STAR-CCM+ (v17.06, Siemens PLM Software Inc., Plano, TX, USA). In total, 20 high-resolution simulations were conducted. The general simulation setup followed the recommendations reported by Berg et al. [22], while the settings for simulations including the CNS were adopted from Korte et al. [17]. The spatial discretization consists of polyhedral cells with a base size of 0.1 mm. In the region around the CNS, the mesh was locally refined to better resolve the strut geometry, with a minimum cell size of 0.02 mm at the CNS struts. Previous investigations using comparable simulation settings demonstrated that this mesh resolution provides mesh-independent hemodynamic results, supporting its application in the present study [17,22].

Total cell counts ranged from 1.06 million (IA5_Post) to 2.3 million (IA2_Pre) for configurations without CNS, and from 4.01 million (IA5_Post) to 8.5 million (IA2_Pre) for configurations including CNS.

Patient-specific inflow measurements were not available. Therefore, an inlet flow waveform reported by Durka et al. [23] and obtained from Doppler ultrasound measurements was used. To ensure consistency and comparability, the same inflow waveform was applied to all investigated configurations.

The outlet boundary conditions were prescribed using a flow-splitting approach based on Murray’s law with a power exponent of *n* = 2, taking the respective outlet cross-sectional areas into account. Previous studies have demonstrated that this approach provides an accuracy comparable to more advanced flow-splitting methodologies [20,24]. All vessel walls were assumed to be rigid, and the flow was considered laminar.

Blood was modeled as an incompressible, non-Newtonian fluid using the Carreau–Yasuda viscosity model according to Karimi et al. [25], with a constant density of 1055 $\mathrm{kg}/m^{3}$.

For each of the investigated configurations, three cardiac cycles were simulated. The first two cycles served as initialization, while the third cycle was used for the hemodynamic evaluation to ensure cycle-independent results.

Simulations were performed on a high-performance computing cluster using 8 computational nodes with 16 CPU cores per node (128 CPU cores in total). Depending on the individual model complexity and mesh size, the computational runtime ranged from approximately 4 to 10 h per simulation. Convergence of the numerical solution was assessed based on residual monitoring, with a continuity convergence criterion of $1\times 10^{-4}$.

### 2.4. Morphological and Hemodynamic Analysis

For the morphological analysis, in addition to the qualitative assessment of the vascular geometries, the ostium area was quantified. Furthermore, the CNS/neck ratio was calculated based on the selected CNS size and the maximum neck width, while the CNS/dome ratio was derived from the selected CNS size and the maximum aneurysm dome width.

The ostium was defined based on the semi-automatic extraction approach described in the previous Section 2.2. The resulting ostium surface was used as the reference surface for the calculation of inflow-related parameters. For the parent vessel reference, a vessel segment located proximal to the aneurysm along the inflow path was selected. This parent artery segment was consistently defined at the same anatomical location in both the pre- and post-interventional configurations to ensure comparability between the two configurations. For the post-processing of the hemodynamics, three flow and three shear parameters were evaluated. In the post-interventional configurations, the IA region refers to the region above the ostium, since this region is not always fully preserved after CNS deployment. The definition of the parameters is as follows:

- TAv: The temporally and spatially averaged velocity within the aneurysm.
- NIR (Neck Inflow Rate): The flow rate entering the aneurysm through the ostium, calculated as:
  (1) $$NIR=\int_{A_{OIA}}\left(\vec{v}\cdot \vec{n}\right)\;dA$$
  where $A_{OIA}$ denotes the ostium inflow area, i.e., the portion of the ostium surface with flow entering the aneurysm. $\vec{v}$ represents the local blood flow velocity vector and $\vec{n}$ denotes the normal vector of the ostium surface.
- ICI (Inflow Concentration Index): The degree of flow concentration entering the aneurysm through the ostium, calculated as:
  (2) $$ICI=\frac{NIR/Q_{parent}}{A_{OIA}/A_{ostium}}$$
  where $Q_{parent}$ represents the flow rate in the reference parent vessel proximal to the aneurysm, $A_{OIA}$ denotes the ostium inflow area, and $A_{ostium}$ denotes the total ostium surface area.
- AWSS (Averaged Wall Shear Stress): Time-averaged tangential shear stress along the aneurysm wall.
- nWSS (Normalized Wall Shear Stress): Ratio between the spatially averaged AWSS within the aneurysm and the corresponding spatially averaged AWSS of the parent vessel.
- HSA (High Shear Area): Percentage of the aneurysm surface area exposed to abnormally high wall shear stress. Abnormally high WSS was defined as values exceeding one standard deviation above the mean AWSS of the reference parent vessel. The HSA was calculated as the ratio between the aneurysm wall area exceeding this threshold and the total aneurysm wall area.

In addition, following the approach of Korte et al. [12], the treatment effect (TE) of the CNS was calculated as the percentage difference between the configuration without CNS and the configuration with CNS deployment, according to the following equation:

(3) $$TE=\frac{{\mathrm{Parameter}}_{\mathrm{with}\;\mathrm{CNS}}-{\mathrm{Parameter}}_{\mathrm{without}\;\mathrm{CNS}}}{{\mathrm{Parameter}}_{\mathrm{without}\;\mathrm{CNS}}}\times 100$$

Given the limited number of included cases, formal statistical analyses were not performed, as the resulting statistical power would be insufficient and could lead to unreliable interpretations. Therefore, the findings were assessed using descriptive analyses.

## 3. Results

### 3.1. Morphological Changes in the Neck Region

The morphological changes are shown in Figure 1. All five aneurysms exhibit a noticeable reduction in contrast enhancement in the dome region. In all post-interventional configurations, the aneurysm dome is no longer identifiable due to the deployment of the CNS device. In IA2 and IA4, the shape of the CNS can be distinctly recognized, as no contrast agent is present distal to the device, thereby explaining the observed morphology. In IA3, the original aneurysm dome is more clearly preserved than in the other cases. While the CNS induces a noticeable deformation, the bleb observed in the pre-interventional configuration is still present, indicating ongoing contrast filling in that region.

Across all configurations, a noticeable change in the neck region can be recognized, with an overall widening of the neck area. Therefore, the variations in the ostium area are presented in Table 2. A clear enlargement of the ostium surface area is observed in all post-interventional configurations, despite using an identical ostium definition and the same extraction procedure for the pre-interventional and post-interventional configurations. The increase is particularly pronounced in IA1, where the post-interventional ostium area is approximately 200% larger than the pre-interventional area.

Table 2 also presents the CNS/neck and CNS/dome ratios, which relate the CNS device size to the morphological characteristics relevant for CNS size selection. For the CNS/neck ratio, it can be observed that the largest ratio, corresponding to the greatest deviation from the nominal CNS range, is associated with the highest percentage change. In contrast, this trend is not observed for the CNS/dome ratio. Here, the largest ratio occurs at the second smallest percentage change.

A further qualitative observation is that the surrounding parent vessels show minimal changes between the pre-interventional and post-interventional configurations, and the overall curvature profiles remain largely preserved.

### 3.2. Hemodynamic Changes

#### 3.2.1. Qualitative Results

**Figure 2:**
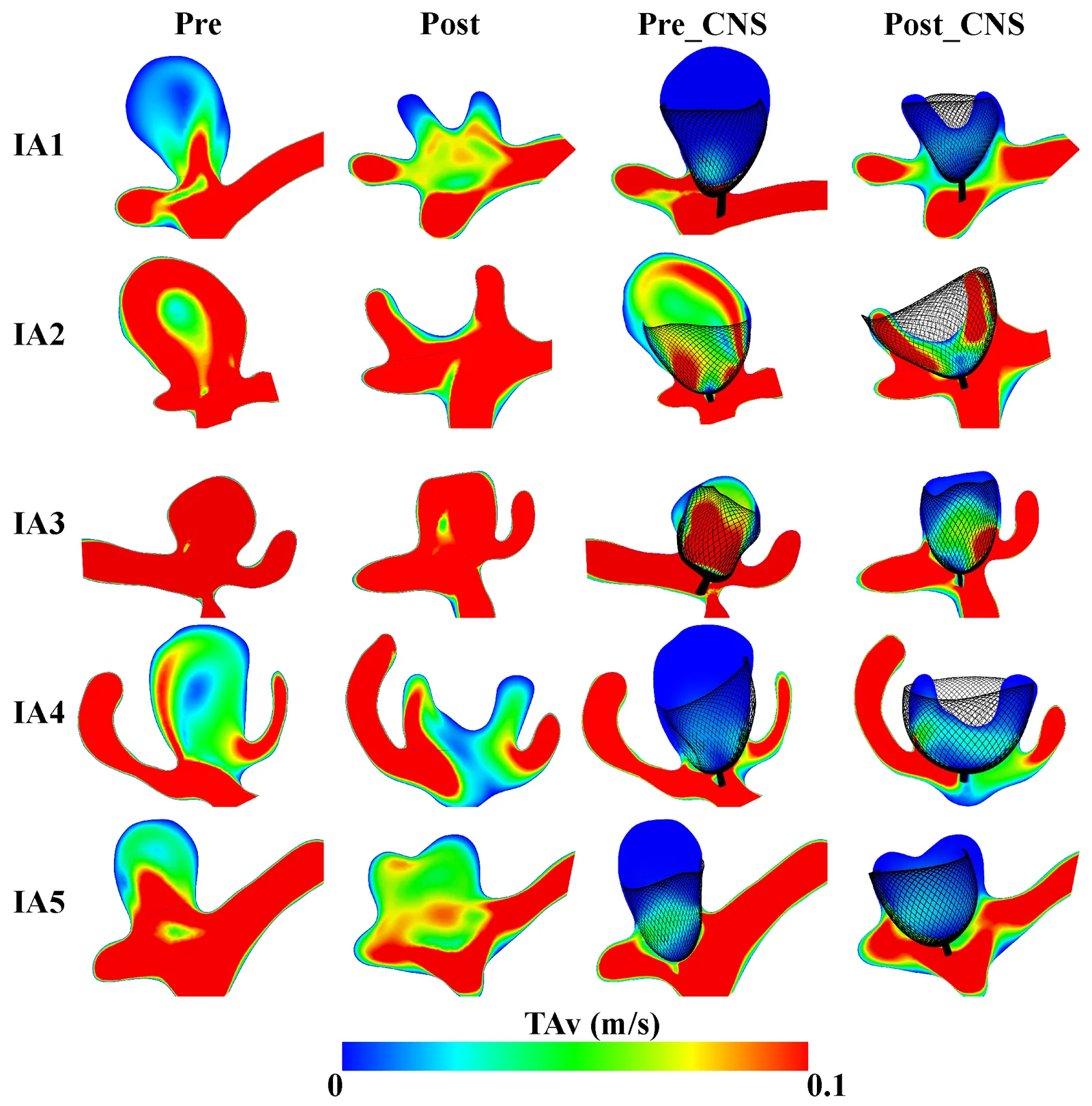
Qualitative representation of the flow-related parameter TAv (time-averaged velocity) across all IA cases in pre-interventional and post-interventional configurations, visualized using a cross-sectional view through the aneurysm region.

Figure 2 shows the qualitative flow results using cross-sectional views through the aneurysm for all cases. Overall, CNS deployment results in a reduction of flow velocity within the IA, or, in the post-interventional configurations, within the region corresponding to the original IA location.

This reduction is particularly evident in the post-interventional configurations IA4 and IA5, where a clear decrease in intra-aneurysmal velocities can be observed. In contrast, case IA2 does not demonstrate a similarly pronounced reduction, with comparatively higher velocities persisting within the IA. It should, however, be noted that this case exhibits generally higher velocities compared with the other IAs.

**Figure 3:**
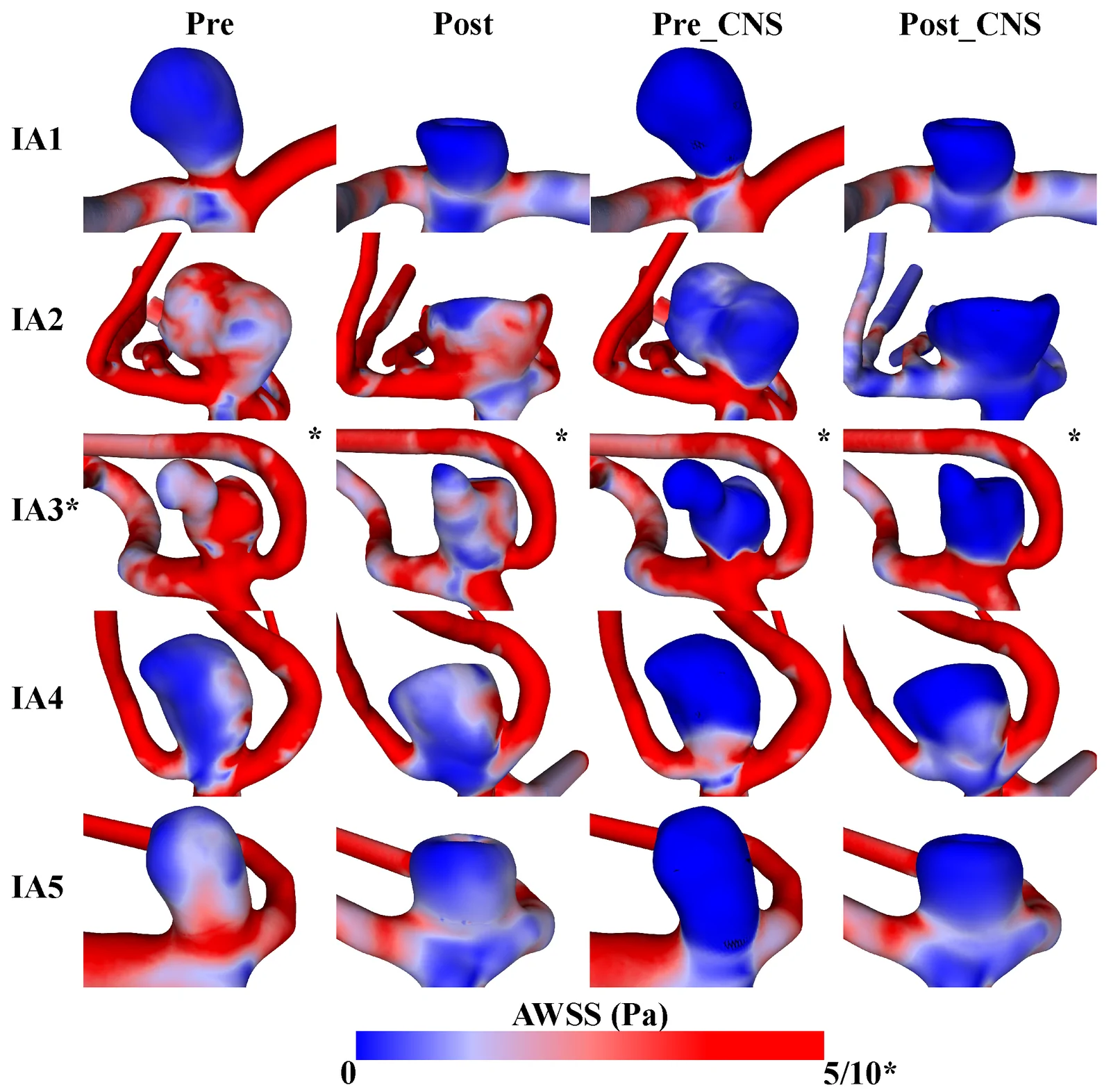
Qualitative representation of the shear-related parameter AWSS (time-averaged wall shear stress) across all IA cases in pre-interventional and post-interventional configurations (* the scale was adjusted for IA3 due to higher values).

The qualitative AWSS distributions for all cases and configurations are shown in Figure 3. Across all cases, a marked reduction in AWSS is observed when comparing configurations without CNS to those with CNS. This effect is particularly pronounced in cases IA2 and IA3, where regions of elevated AWSS are clearly present in the untreated configurations. Following CNS deployment, these high-AWSS regions are no longer observed within the aneurysm region distal to the ostium.

When comparing pre-interventional and post-interventional configurations without CNS, different patterns are observed. However, due to substantial geometric differences between cases, no definitive conclusion can be drawn as to whether the pre-interventional or post-interventional configuration is associated with higher AWSS.

#### 3.2.2. Quantitative Results

**Figure 4:**
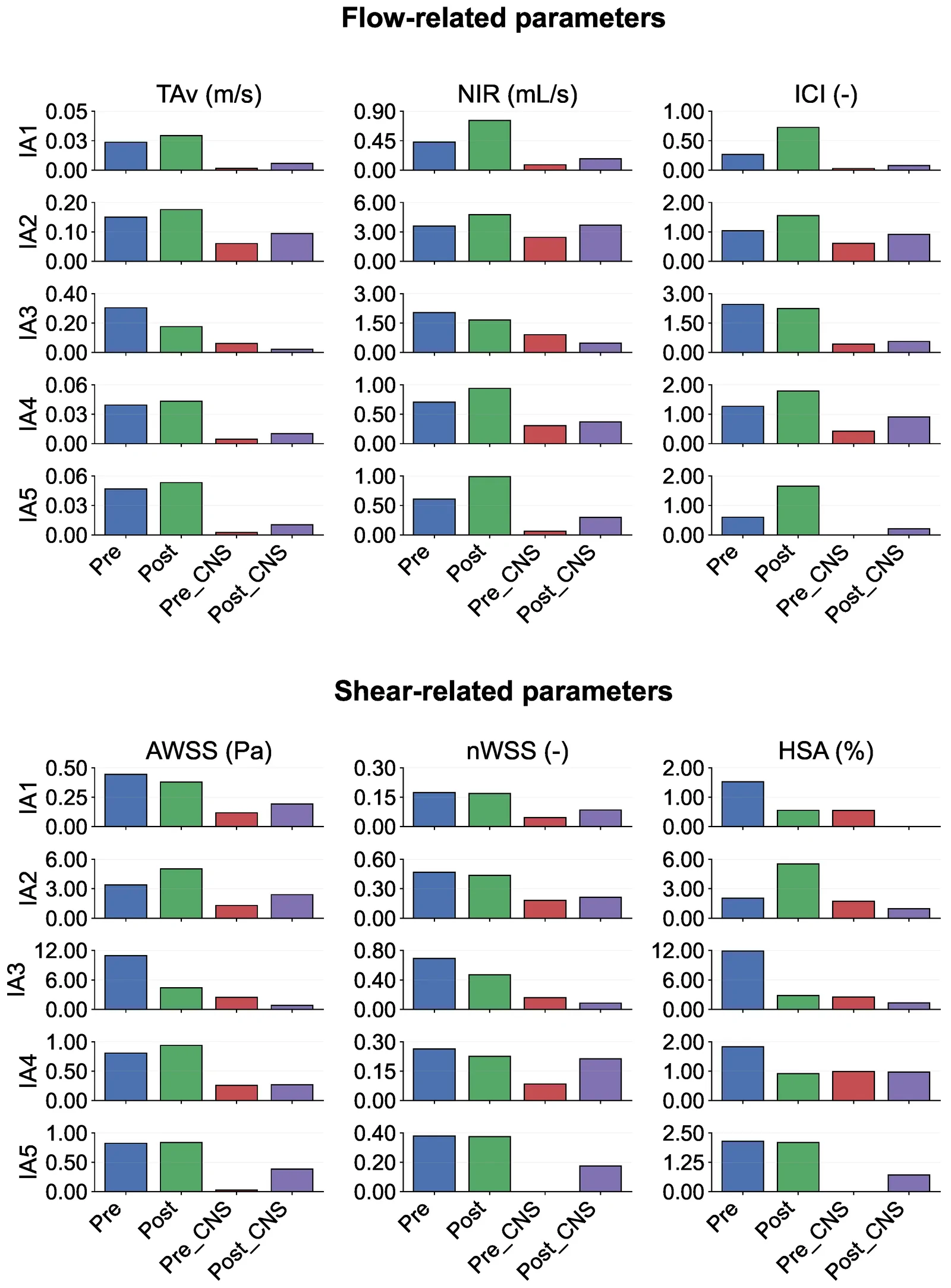
Comparison of flow-related parameters (TAv: time-averaged velocity; NIR: neck inflow rate; ICI: inflow concentration index) and shear-related parameters (AWSS: time-averaged wall shear stress; nWSS: normalized wall shear stress; HSA: high shear area) for pre- and post-interventional configurations without virtual CNS deployment (Pre, Post) and with virtual CNS deployment (Pre_CNS, Post_CNS). The y-axis scales were individually selected for each case and parameter to appropriately represent the respective data ranges and visualize differences between the configurations.

The quantitative results of the evaluated parameters are summarized in the bar charts shown in Figure 4. A consistent reduction is observed for both flow-related and shear-related parameters following CNS deployment, independent of the configuration.

For simulations without CNS, the post-interventional configurations (with the exception of IA3) exhibit higher values for the flow-related parameters. In contrast, the shear-related parameters show less consistent behavior. The HSA is markedly higher in the pre-interventional configuration in four out of five cases, with IA2 representing the only exception. Similarly, nWSS tends to be slightly higher in the pre-interventional configurations across all cases. For AWSS, the results are more heterogeneous: in four aneurysms, slightly higher values are observed in the post-interventional configuration, whereas in the remaining case, higher values occur in the pre-interventional configuration.

Overall, flow velocities within the aneurysms are noticeably lower in cases IA1, IA4, and IA5 compared to IA2 and IA3.

In addition to Figure 4, the percentage differences of the hemodynamic parameters are presented in Table 3. These analyses include the overall differences between the pre-interventional and post-interventional configurations, as well as two treatment effects (TEs). The first TE (TE_Pre) represents the hemodynamic difference between the pre-interventional configuration without CNS and the corresponding pre-interventional configuration with a virtually deployed CNS. The second TE (TE_Post) represents the hemodynamic difference between the post-interventional configuration without CNS and the corresponding post-interventional configuration with CNS. In addition, configuration-induced differences (CID) were assessed to quantify the deviation in the estimated hemodynamic treatment response when hemodynamic parameters derived from the pre-interventional configuration without a virtually deployed CNS were compared with those derived from the final post-interventional configuration with a virtually deployed CNS (CID was computed using the same formulation as TEs (Equation (3))).

The “Post” percentage changes support the findings shown in Figure 4, indicating that flow-related parameters increase in all post-interventional configurations except for IA3. For shear-related parameters, a percentage reduction between the pre-interventional and post-interventional configurations is observed for nWSS and HSA, except for HSA in IA2. In contrast, AWSS does not exhibit a clear trend.

Regarding the treatment response, both TE_Pre and TE_Post demonstrated substantial reductions across the evaluated hemodynamic parameters, indicating a consistent hemodynamic response to CNS deployment across both configurations. Differences between TE_Pre and TE_Post were observed in a parameter- and case-dependent manner. The CID further quantified the differences between the hemodynamic response obtained from pre-interventional and post-interventional treated configurations, with lower hemodynamic reductions observed compared with TE_Pre for most aneurysms and parameters.

## 4. Discussion

The CNS represents a promising treatment option for complex wide-neck bifurcation aneurysms [26]. However, as a relatively new device, clinical follow-up data remain limited, and in silico studies are still scarce [27], while existing studies suggest that the device achieves the intended flow-modulating effect [12,17,28], the exact relationship between CNS-associated morphological changes and their subsequent impact on hemodynamics has not yet been systematically investigated. The present pilot study aims to address this gap by analyzing these morphological–hemodynamic interdependencies in detail.

### 4.1. Morphological Changes

The morphological analysis revealed a clear expansion of the neck region, reflected by an increase in the ostium area. Spitz et al. [21] investigated this parameter as well. They reported an increased ostium area in three out of seven aneurysms, whereas the area decreased again in the remaining cases. However, their study was based on follow-up data, whereas the present study analyzed 24 h post-interventional data. In the present study, only 24 h post-interventional data were used for the analysis. Therefore, the findings suggest that neck expansion was observed 24 h after treatment, although modality- and segmentation-related effects cannot be fully excluded. This expansion may partially regress over time during the healing process, particularly in association with endothelialization of the aneurysm sac. Although follow-up data in this case series were limited in quality and availability, visual assessment indicated that the ostium area remained larger than in the pre-interventional configuration in all five aneurysms. Collectively, these findings suggest that an enlarged neck region was observed at 24 h post-intervention and may decrease during subsequent healing, as reported by Spitz et al. [21]. However, this hypothesis warrants further investigation and remains difficult to evaluate due to heterogeneous follow-up time points, ongoing healing processes at different stages, and the still incompletely understood mechanisms of aneurysm healing [29,30]. Therefore, analysis of the hemodynamic conditions at 24 h post-intervention is particularly valuable, as these data may provide insights into early hemodynamic changes potentially associated with subsequent treatment response.

Another important observation from the morphological analysis is that the surrounding vessels did not exhibit immediate morphological differences following CNS deployment. In contrast, flow diverters or stent-assisted coiling may induce vessel straightening or vessel remodeling, as reported by Voß et al. [13], Leng et al. [31], and Lu et al. [32]. This effect was not observed with the CNS device; instead, the observed morphological changes were primarily limited to the neck region.

The interpretation of the observed morphological changes requires consideration of the different imaging modalities used for the pre-interventional and post-interventional configurations, while 3D-DSA was used for the pre-interventional configuration, CTA was applied for the post-interventional configurations, reflecting common clinical practice due to its lower invasiveness and broader availability. However, CTA is associated with lower spatial resolution and increased susceptibility to partial-volume effects, which may particularly affect segmentation of complex regions such as the aneurysm neck [33]. Previous studies have demonstrated that small geometric differences between CTA- and 3D-DSA-derived aneurysm models are expected, while overall agreement remains high. Ren et al. [34] reported mean surface distances of approximately 0.25±0.07 mm between CTA- and 3D-DSA-based vascular models [34]. The additional surface comparison presented in Appendix A.2 demonstrated similarly low deviations across all cases (MSD: 0.26±0.06 mm; RMSD: 0.33±0.08 mm), indicating that the reconstructed geometries are within the range of previously reported inter-modality differences. Although segmentation-related uncertainties, particularly at the aneurysm neck, cannot be excluded, the observed increase in ostium area exceeded the magnitude of the expected geometric deviations. Nevertheless, the contribution of imaging modality and segmentation to the observed ostium enlargement cannot be completely ruled out. Future studies including matched pre-interventional and post-interventional 3D-DSA acquisitions are required to further validate these findings.

A further interesting aspect of the observed neck enlargement is that the underlying mechanisms remain unclear, while mechanical deformation caused by the implanted device could contribute to this morphological change, biological processes such as thrombus formation may also influence the post-treatment geometry. Current in silico approaches typically neglect these biological processes and mainly evaluate the immediate hemodynamic effect of device implantation. Future studies incorporating thrombus formation models could provide further insights into the temporal evolution of aneurysm remodeling and treatment response. Such approaches have recently been explored for flow-diverter treatments by modeling fibrin accumulation and thrombus-related flow alterations [35].

### 4.2. Device Size and Deformation

Along with the morphological analysis, the CNS/neck and CNS/dome ratios were calculated. An interesting observation was that the largest CNS/neck ratio coincided with the greatest change in ostium size. This finding suggests a possible association between device size and morphological changes, potentially related to the mechanical forces exerted by larger devices. In contrast, no clear relationship between the CNS/dome ratio and the extent of morphological changes was observed. Similar findings have been reported by Larsen et al. [5], who found no significant association between the CNS/neck ratio and device deformation. Likewise, neither oversizing nor undersizing was associated with recurrence or angiographic outcomes. Nevertheless, the authors emphasized that optimal device sizing remains an important consideration. To date, sizing strategies for the CNS have only been investigated in a limited number of experimental studies, none of which incorporated post-interventional data [36], while clinical practice generally favors slight oversizing [5]. Such recommendations are also based on experience with other intrasaccular flow-disrupting devices. For example, oversizing of the Woven EndoBridge (WEB) Embolization System (MicroVention/Terumo Neuro, Aliso Viejo, CA, USA) has been associated with improved angiographic outcomes [37].

However, device-specific sizing recommendations for the CNS that account for post-interventional morphological changes have not yet been systematically evaluated. Future studies should focus on establishing dedicated sizing strategies, for example by defining a specific sizing factor, while considering CNS-associated differences. Such investigations may help clarify the relationship between device size and morphological alterations and identify aneurysm geometries that are most suitable for CNS treatment.

In addition, the visualizations in Figure 1 illustrate that the virtual CNS devices require adaptation between the pre-interventional and post-interventional configurations. While this does not represent the idealized deployment of the medical device, the observed morphological differences between the configurations suggest that the CNS may undergo a certain degree of deformation during implantation. Device deformation has also been reported in previous clinical studies [38,39], and Larsen et al. [5] associated such deformation with potential recurrence and unstable aneurysm occlusion. Based on these findings, Larsen et al. [5] recommended the use of three-dimensional follow-up imaging to better assess device deformation over time. In line with this, the morphological and hemodynamic changes observed in the present study further highlight the potential value of follow-up imaging for a more comprehensive evaluation of post-interventional morphological and hemodynamic changes after CNS implantation.

### 4.3. Hemodynamic Changes

Both qualitative and quantitative analyses indicate that CNS deployment consistently leads to a reduction in hemodynamic parameters, regardless of whether the pre-interventional or post-interventional configuration is considered. This finding was expected and is consistent with previous in silico studies [12,17] and clinical investigations [2,3]. The comparison between pre-interventional and post-interventional configurations without CNS does not represent a realistic clinical scenario, as the latter configuration cannot occur in clinical practice. Nevertheless, this comparison provides valuable insight into whether the observed morphological changes may affect hemodynamic conditions.

From a hemodynamic perspective, clear differences are observed between the pre-interventional and post-interventional configurations. Except for one IA case, the post-interventional configurations consistently show higher intra-aneurysmal velocities, accompanied by increased NIR and ICI values. These changes coincide with the observed enlargement of the ostium area and suggest that the altered geometry may allow a greater amount of blood to enter the aneurysm while maintaining a more concentrated inflow pattern. Consequently, the intra-aneurysmal flow pattern appears to remain predominantly jet-dominated rather than being redistributed more homogeneously throughout the aneurysm sac. Interestingly, despite these changes, the pre-interventional configurations exhibited a larger proportion of HSA, indicating that increased inflow in the post-interventional configurations does not necessarily translate into a more extensive distribution of elevated AWSS.

### 4.4. Treatment Effect and Outcome

In silico studies can serve as a valuable tool for evaluating endovascular devices and may help predict treatment outcomes or retrospectively interpret observed results. However, such studies often rely on simplifying assumptions or are limited to pre-interventional configurations. To assess the impact of relying solely on pre-interventional configurations for in silico assessment of CNS-associated hemodynamic changes, two treatment effect metrics were calculated to evaluate the hemodynamic response to CNS deployment under different configurations. TE_Pre and TE_Post both demonstrated substantial reductions in hemodynamic parameters, indicating a consistent hemodynamic response to CNS deployment across both configurations. However, differences between TE_Pre and TE_Post were observed depending on the aneurysm and the evaluated hemodynamic parameter. To further quantify the impact of using pre-interventional configurations instead of the final post-interventional configuration, the CID was calculated. The observed CID values suggest that CNS-associated morphological changes can modify the estimated hemodynamic response and may need to be considered when interpreting in silico treatment evaluations.

Whether these differences in TE translate into clinically relevant changes remains unclear due to the limited clinical evidence currently available for the CNS. Ouared et al. [40] reported that, for flow diverters, a reduction in intra-aneurysmal velocity greater than 35% is associated with successful aneurysm occlusion. In the present study, this threshold was achieved for the TAv in all investigated configurations and TE calculations, indicating that despite differences between TE_Pre and TE_Post, all simulations predicted a hemodynamic response above this previously reported threshold. However, whether similar hemodynamic thresholds exist for CNS treatment remains unknown and warrants further investigation in future studies.

An additional observation in this pilot study is that the aneurysm with the lowest treatment effects (TE_Pre and TE_Post) also showed subsequent deterioration in outcome (IA2). However, given the small sample size and the occurrence of only a single event, this finding should be interpreted as hypothesis-generating rather than predictive. Notably, this case was characterized by relatively elevated pre-interventional flow velocities, suggesting that hemodynamic conditions may contribute to treatment response. Previous studies on the WEB device have also reported associations between flow-related parameters and angiographic outcomes [41,42]. Future studies in larger and more homogeneous cohorts are needed to evaluate the predictive value of numerical simulations and to further clarify the role of morphological and hemodynamic parameters in treatment outcomes. Data-driven approaches, including machine learning, may complement physics-based modeling; however, their clinical applicability remains to be established [43]. From a clinical perspective, these findings emphasize the relevance of considering post-interventional morphology in computational assessments. Incorporating follow-up imaging may help to better capture treatment-related anatomical changes and support future patient-specific computational approaches.

Overall, successful CNS implantation appears to be a multifactorial process, in which morphological changes, hemodynamic alterations, and device deformation are closely interconnected and warrant further investigation in future studies. Improved understanding of these interactions may also inform treatment strategies, such as the potential role of adjunctive coiling in selected aneurysms, with the aim of achieving more stable and effective occlusion outcomes [44,45]. Differences between pre-interventional and post-interventional configurations may influence the estimated hemodynamic response, suggesting that follow-up imaging could improve the patient-specific evaluation of treatment effects. In the future, such approaches may support more reliable computational treatment planning and monitoring of treatment response.

### 4.5. Limitations

First, a limitation of this retrospective study is the small case series. Although consistent trends in morphological changes were observed, the limited sample size did not permit robust statistical analyses. Therefore, these findings should be confirmed in a larger population to strengthen their generalizability and to enable robust statistical analyses of potential relationships between hemodynamic parameters, morphological changes, and treatment outcomes. Second, uncertainty is introduced by the reconstruction of post-interventional configurations and the associated segmentation challenges. The use of different imaging modalities for the pre- and post-interventional configurations may influence the derived aneurysm morphology due to modality-dependent differences in spatial resolution, contrast, and susceptibility to segmentation errors. Although additional geometric analyses demonstrated high agreement between the reconstructed models, small modality-related differences, particularly in complex regions such as the aneurysm neck, cannot be excluded. Additionally, the reproducibility of extracted ostium measurements was not formally assessed and represents a limitation. Furthermore, reconstruction of post-interventional aneurysm geometries remains challenging due to incomplete contrast filling and the absence of a standardized reconstruction approach for treated aneurysms. While the present study applied a strategy aimed at minimizing artificial geometric modifications, alternative reconstruction approaches may be more suitable depending on the specific research question and require further investigation. Third, the virtual CNS deployment was performed manually, introducing a degree of operator dependency. The virtual CNS deployment followed the workflow described by Korte et al. [12], which was previously evaluated using complementary in vitro and in silico experiments. In addition, the simulated device configurations were reviewed with the treating neuroradiologist to ensure clinical plausibility. Nevertheless, patient-specific validation against post-interventional imaging and a formal assessment of operator-dependent variability were not performed in the present study and represent limitations of the current approach. Furthermore, the exact implanted CNS geometry could not be reconstructed from the available imaging data. Therefore, the virtual device configuration represents an approximation of the in vivo implant state. Differences between the virtual and actual device configurations, including variations in device position, deformation, and the extent of neck coverage, may influence the resulting hemodynamic parameters. Although virtual deployment approaches are widely used in computational studies of endovascular devices, they cannot fully reproduce the complex interaction between the device and the vessel wall occurring during implantation. Fourth, the simulations relied on established simplifications, including rigid vessel walls and generic boundary conditions, which may affect absolute hemodynamic values. However, identical settings across all cases allowed consistent comparison of relative changes between pre-interventional and post-interventional configurations. Future studies incorporating patient-specific boundary conditions may further improve physiological representation. Finally, thrombus formation was not explicitly modeled. Instead, the post-interventional configurations were analyzed as reconstructed from follow-up imaging, while this approach enables assessment of the observed post-interventional configurations, future models incorporating thrombus evolution may provide additional insights into the mechanisms underlying aneurysm healing and treatment outcomes. Future studies including larger multicenter cohorts are needed to validate the observed trends. Furthermore, incorporating patient-specific boundary conditions may improve the physiological representation of the hemodynamic environment and help to further assess the clinical relevance of these findings.

## 5. Conclusions

Comparison of pre-interventional and post-interventional configurations revealed morphological differences, most notably alterations of the aneurysm neck and an increase in ostium area. In this pilot study, these changes were accompanied by variations in intra-aneurysmal hemodynamics and may influence the estimated treatment effect when analyses rely solely on pre-interventional configurations with virtually deployed devices. The results indicate that treatment outcomes are likely influenced by a complex interplay of morphological changes, flow alterations, and potential device-related deformation. These preliminary observations underscore the importance of a combined assessment of morphological and hemodynamic factors in post-interventional evaluation. Confirmation in larger cohorts is necessary to establish reliable predictors of treatment outcome, while three-dimensional follow-up imaging may enhance the characterization of these complex interactions.

## Figures and Tables

**Table 1:** Overview of patient characteristics (age and sex), aneurysm-specific parameters (location and rupture status), morphological features (aneurysm volume (IA Volume), maximum neck width (IA Neck), and maximum dome width (IA Dome)), and treatment outcomes (CNS size and occlusion status).

Case	Patient		Aneurysm		Morphology			Treatment	
	Sex	Age (y)	Location	Rupture	IA Volume (mm^3^)	IA Neck (mm)	IA Dome (mm)	CNS Size (mm)	Complete Occlusion
IA1	m	57	ICA	no	64.1	2.5	5.0	7	yes
IA2	f	47	MCA	no	394.02	4.2	9.0	11	no
IA3	f	71	ACOM	no	29.49	2.9	3.9	7	yes
IA4	f	50	ACOM	no	114.5	4.3	5.4	9	yes
IA5	f	54	ACOM	no	29.53	3.2	3.5	7	yes

Abbreviations: m, male; f, female; ICA, internal carotid artery; MCA, middle cerebral artery; ACOM, anterior communicating artery.

**Table 2:** Comparison of ostium area before (Pre_Area) and after CNS implantation (Post_Area), including the percentage change between the two configurations. Additionally, the CNS/neck and CNS/dome ratios are provided, describing the relationship between the selected device size and the morphological characteristics of the individual aneurysms.

Case	Pre_Area (mm^2^)	Post_Area (mm^2^)	Percentage Change (%)	CNS/Neck Ratio (-)	CNS/Dome Ratio (-)
IA1	4.44	13.36	200.90	2.80	1.40
IA2	16.58	34.36	107.24	2.62	1.23
IA3	5.30	8.64	63.02	2.41	1.79
IA4	9.70	24.18	149.28	2.09	1.67
IA5	8.53	14.75	72.92	2.19	2.0

**Table 3:** Percentage changes in flow-related parameters (TAv: time-averaged velocity; NIR: neck inflow rate; ICI: inflow concentration index) and shear-related parameters (AWSS: time-averaged wall shear stress; nWSS: normalized wall shear stress; HSA: high shear area) between pre- and post-interventional configurations (Post), treatment effects for pre-interventional (TE_Pre: pre without CNS vs. pre with CNS) and post-interventional (TE_Post: post without CNS vs. post with CNS) configurations, and configuration-induced differences (CID: pre without CNS vs. post with CNS) for IA1–IA5.

IA	State	TAv	NIR	ICI	AWSS	nWSS	HSA
1	Post	23.82%	76.97%	172.97%	−14.66%	−2.77%	−63.63%
	TE_Pre	−93.71%	−81.02%	−89.77%	−73.46%	−73.42%	−63.91%
	TE_Post	−80.25%	−77.08%	−89.23%	−49.23%	−49.73%	−100%
	CID	−75.55%	−59.44%	−70.62%	−56.68%	−56.68%	−100%
2	Post	16.98%	33.10%	49.28%	49.14%	−6.28%	170.10%
	TE_Pre	−59.92%	−31.89%	−41.47%	−61.57%	−28.44%	−18.09%
	TE_Post	−46.17%	−22.96%	−41.56%	−52.37%	−51.46%	−82.45%
	CID	−37.02%	2.54%	−12.77%	−28.97%	−54.49%	−52.55%
3	Post	−42.12%	−18.67%	−8.55%	−59.88%	−32.26%	−67.63%
	TE_Pre	−79.98%	−55.32%	−82.34%	−77.50%	−77.25%	−78.81%
	TE_Post	−88.23%	−71.35%	−74.92%	−82.01%	−81.69%	−65.53%
	CID	−93.18%	−76.69%	−77.06%	−92.78%	−87.58%	−88.84%
4	Post	10.02%	32.82%	40.74%	16.23%	−14.66%	−49.98%
	TE_Pre	−88.76%	−56.72%	−66.31%	−68.21%	−67.93%	−46.27%
	TE_Post	−76.21%	−60.59%	−49.09%	−71.41%	−5.43%	5.64%
	CID	−73.88%	−47.67%	−28.35%	−66.77%	−19.30%	−47.16%
5	Post	13.79%	62.39%	175.82%	1.60%	−1.17%	−13.25%
	TE_Pre	−94.90%	−90.08%	−93.35%	−96.75%	−96.69%	−100.00%
	TE_Post	−80.53%	−69.87%	−87.38%	−54.03%	−53.52%	−65.85%
	CID	−77.84%	−51.06%	−65.19%	−55.30%	−56.06%	−70.38%

**Figure A1:**
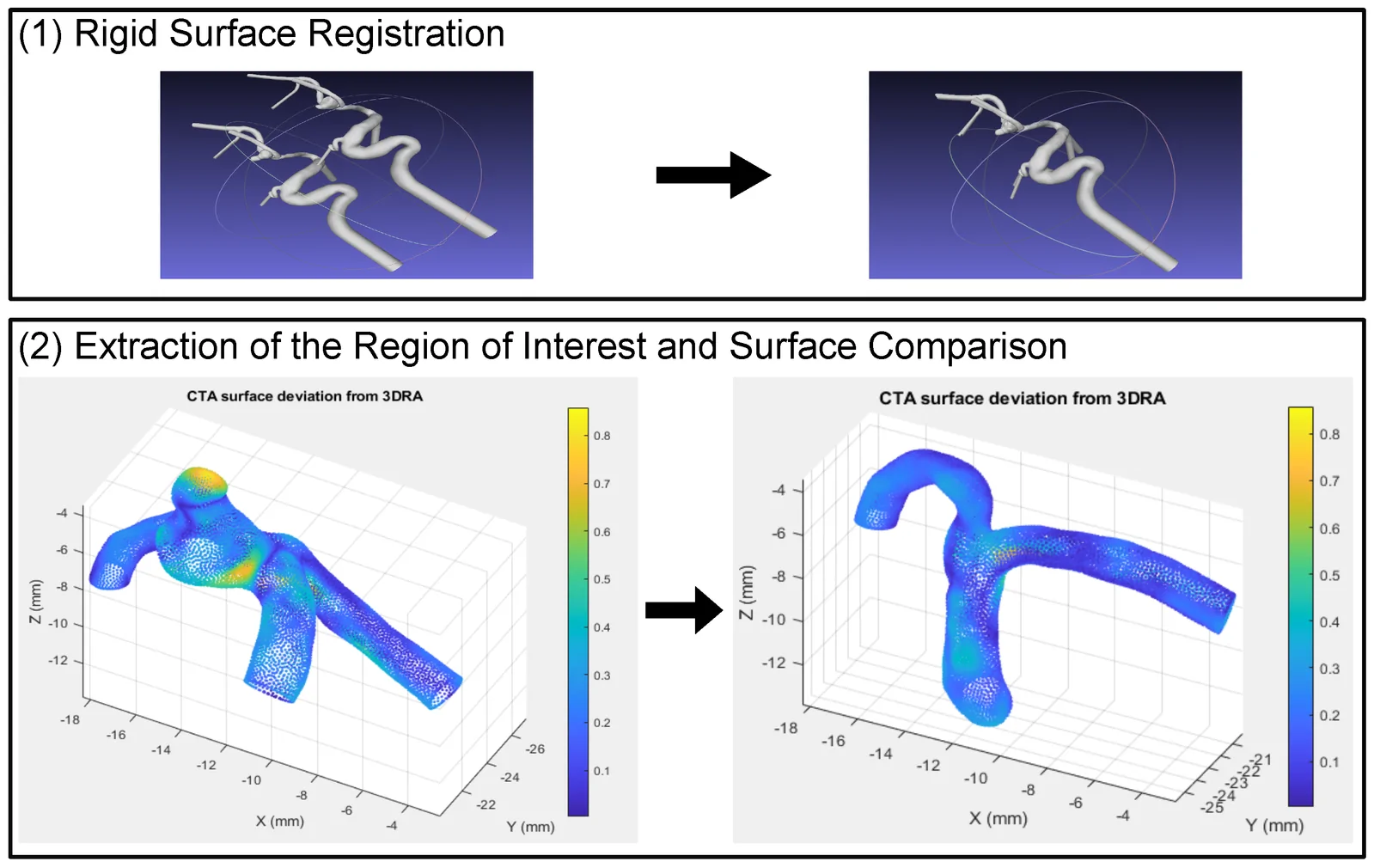
Workflow (**1**) Rigid surface registration of the CTA- and 3D-DSA-derived vascular models performed in MeshLab. (**2**) Surface-based comparison of the aligned models, including the complete vascular geometry (left) and the cropped region of interest excluding the aneurysm sac (right).

## Data Availability

Data are available upon reasonable request.

## Abbreviations

The following abbreviations are used in this manuscript:

3D-DSA	Three-dimensional digital subtraction angiography
CID	Configuration-induced difference
CNS	Contour Neurovascular System
CTA	Computed tomography angiography
HSA	High shear area
IA	Intracranial aneurysm
ICI	Inflow concentration index
MSD	Mean surface distance
NIR	Neck inflow rate
RMSD	Root mean square distance
STL	Standard tessellation language
TAv	Time-averaged velocity
TE	Treatment effect
WEB	Woven EndoBridge Embolization System
WSS	Wall shear stress

## Appendix A

### Appendix A.1. Device Selection and Sizing Criteria for CNS Deployment

The following Table A1 summarizes the manufacturer’s sizing recommendations for CNS selection based on aneurysm dimensions [46]. Device diameter was selected according to the maximum aneurysm dome width and neck diameter, measured from the pre-interventional 3D-DSA reconstruction.

**Table A1:** CNS device sizing recommendations based on aneurysm dimensions, including recommended device diameters according to maximum dome width and aneurysm neck width. Adapted from the manufacturer’s specifications provided by Stryker Neurovascular [46].

Device Size [mm]	Max. Aneurysm Dome Width * [mm]	Max. Aneurysm Neck [mm]
5.0	2.0–3.5	2.0–3.0
7.0	3.0–5.5	3.0–5.0
9.0	5.0–7.5	4.0–6.0
11.0	7.0–8.5	5.0–8.0
14.0	8.0–10.5	7.0–10.0

* Maximum dome width is equivalent to equatorial diameter.

### Appendix A.2. Assessment of Geometric Differences Between Imaging Modalities

In clinical practice, different imaging modalities are frequently used for pre-interventional and post-interventional assessment, as reflected in the present study. To enable a quantitative evaluation of morphological and hemodynamic changes despite differences in image acquisition, the geometric differences between vascular models reconstructed from different imaging modalities were systematically assessed.

The workflow of the geometric comparison is illustrated in Figure A1 for IA3. Since the segmented surface models were reconstructed from different imaging modalities, the resulting standard tessellation language (STL) models were initially defined in different coordinate systems. Therefore, rigid registration was performed in MeshLab to spatially align the models before further analysis.

After registration, the models were cropped to a predefined region of interest (ROI) to exclude unrelated anatomical structures and potential segmentation artifacts that could affect the surface comparison. An initial comparison of the complete anatomical models was subsequently performed. As expected, the largest deviations were observed in the aneurysm region, which were mainly attributed to post-interventional morphological changes of the aneurysm sac. Therefore, an additional analysis was performed after excluding the aneurysm sac region, enabling an isolated assessment of the surrounding parent vessels and the geometric agreement between imaging modalities while minimizing the influence of aneurysm-specific morphological alterations.

To quantify the geometric agreement between STL models derived from different imaging modalities, a bidirectional nearest-neighbor surface distance analysis was performed. For each vertex of the reference surface, the Euclidean distance to the closest point on the corresponding test surface was calculated. The analysis was repeated in the opposite direction (test-to-reference) to account for asymmetric local deviations between the two surfaces. The resulting bidirectional distance distribution was subsequently used to calculate quantitative similarity metrics.

The mean surface distance (MSD) was calculated as the arithmetic mean of all bidirectional nearest-neighbor surface distances:

(A1) $$MSD=\frac{1}{N}\sum_{i=1}^{N}d_{i},$$

where $d_{i}$ represents the Euclidean distance between a surface point and its nearest point on the opposing surface, and *N* denotes the total number of evaluated distances. The MSD describes the average geometric deviation between the two surface models, with lower values indicating higher geometric agreement.

The root mean square distance (RMSD) was calculated as

(A2) $$RMSD=\sqrt{\frac{1}{N}\sum_{i=1}^{N}d_{i}^{2}}.$$

Compared with MSD, RMSD increases the contribution of larger deviations by squaring individual distances before averaging. Therefore, RMSD is more sensitive to localized regions with pronounced geometric discrepancies, while still providing an overall measure of surface similarity between the two models.

The quantitative surface comparison in Table A2 demonstrated small geometric deviations between CTA- and 3D-DSA-derived vascular models, with MSD values ranging from 0.184 to 0.332 mm and RMSD values between 0.218 and 0.450 mm. These differences are within the range of previously reported geometric deviations for intracranial aneurysm models reconstructed from different imaging modalities. Ren et al. reported a mean surface distance of 0.25 ± 0.07 mm between CTA- and 3D-DSA-derived models, demonstrating that modality-related geometric variations in the submillimeter range are expected during patient-specific aneurysm reconstruction [34].

**Table A2:** Quantitative assessment of geometric differences between CTA- and 3D-DSA-derived vascular models. Surface distance metrics, including mean surface distance (MSD) and root mean square distance (RMSD), are reported for each case.

Case	MSD (mm)	RMSD (mm)
IA1	0.228	0.309
IA2	0.332	0.450
IA3	0.184	0.218
IA4	0.264	0.331
IA5	0.299	0.356
